# Iso-Oriented Anatase TiO_2_ Mesocages as a High Performance Anode Material for Sodium-Ion Storage

**DOI:** 10.1038/srep11960

**Published:** 2015-07-06

**Authors:** Zhensheng Hong, Kaiqiang Zhou, Zhigao Huang, Mingdeng Wei

**Affiliations:** 1 Fujian Provincial Key Laboratory of Quantum Manipulation and New Energy Materials, College of Physics and Energy, Fujian Normal University, Fuzhou, Fujian 350108, China; 2 State Key Laboratory of Photocatalysis on Energy and Environment, Fuzhou University, Fuzhou, Fujian 350002, China; 3 Institute of Advanced Energy Materials, Fuzhou University, Fuzhou, Fujian 350002, China

## Abstract

A major obstacle in realizing Na-ion batteries (NIBs) is the absence of suitable anode materials. Herein, we firstly report the anatase TiO_2_ mesocages constructed by crystallographically oriented nanoparticle subunits as a high performance anode for NIBs. The mesocages with tunable microstructures, high surface area (204 m^2^ g^−1^) and uniform mesoporous structure were firstly prepared by a general synthesis method under the assist of sodium dodecyl sulfate (SDS). It’s notable that the TiO_2_ mesocages exhibit a large reversible capacity and good rate capability. A stable capacity of 93 mAhg^−1^ can be retained after 500 cycles at 10 C in the range of 0.01–2.5 V, indicating high rate performance and good cycling stability. This could be due to the uniform architecture of iso-oriented mesocage structure with few grain boundaries and nanoporous nature, allowing fast electron and ion transport, and providing more active sites as well as freedom for volume change during Na-ion insertion. CV measurements demonstrate that the sodium-ion storage process of anatase mesocages is mainly controlled by pseudocapacitive behavior, which is different from the lithium-ion storage and further facilitates the high rate capability.

Developing the rechargeable batteries is critical to address the increasing demand of mobile devices, electric-powered transportation, and stationary energy storage. Lithium-ion batteries (LIBs) have been the most promising battery technology owing to their higher energy density and longer cycle life than other secondary battery systems^1^. However, there is increasing concern about the cost and the limitation of lithium reserves on earth for large-scale applications of LIBs^2^. Sodium is a cheap, abundant and second lightest alkali metal element, and thus has recently attracted great interest for the use as a transporting ion for alternative rechargeable batteries^3^,^4^,^5^. However, the larger diameter of the Na-ion (0.97 Å) compared to Li-ion (0.68 Å) hampers electrochemical reaction kinetics, which makes it difficult to find suitable host materials which have sufficiently large interstitial space to accommodate sodium ions and can allow reversible and rapid ion/electron insertion and extraction^6^.

Now, a major obstacle in realizing Na-ion batteries (NIBs) was the absence of suitable negative electrodes^4^. Graphite was found to be good host material for Li intercalation, and is now commercially utilized as negative electrode materials for LIBs. In the previous study, graphite cannot be utilized as an insertion host of Na ions^6^,^7^. Recently, it has been shown that graphite can be used for sodium-ion batteries by making use of co-intercalation phenomena in spite of the poor rate performance^8^. Some studies of hard carbons as negative materials for NIBs were reported, however, it delivered limited capacity at high current rate^9^,^10^. Recently, some anode materials with alloy-type (Sn and SnO_2_)^11^,^12^ and conversion-type mechanism (CuO and MoS_2_)^13^,^14^ were studied, which showed high initial capacity but suffered from poor cycling performance owing to the large volume change or the sluggish kinetics. Classical insertion materials such as Na_2_Ti_3_O_7_ was proposed as an alternative anode material operating at a low potential of around 0.3 V vs. Na/Na^+^, however, such a material showed a rather low capacity and poor cycling stability^15^,^16^. Therefore, it is still a challenge to develop appropriate anode materials with both high capacity and long cycling life.

Several types of TiO_2_ polymorphs have been used as promising anode materials for LIBs due to its intrinsic advantages in safety, low cost and good cyclic stability^17^,^18^,^19^,^20^. However, only few reports were reported so far on the behavior of Na ions insertion into TiO_2_. Although Rajh *et al.*^21^ firstly investigated the amorphous TiO_2_ nanotube anode for rechargeable NIBs, they concluded that anatase TiO_2_ is inactive in their NIBs. Most recently, it’s found that the Na-ion storage performance of anatase TiO_2_ nanostructures could be improved by carbon coating or graphene doping^22^,^23^,^24^,^25^. However, there are different opinions about the sodium storage process. Cha *et al.*^24^ and Kim *et al.*^25^ claimed the similar suggestion that sodium ions would be reversibly (de-)inserted into the anatase host observed from the lattice expansion and structure remaining during the intercalation and extraction process. On the contrary, Wu *et al.*^26^ very recently reported that the anatase TiO_2_ would be partly reduced to metallic titanium and amorphous sodium titanate phase which could reversibly store about 0.41 sodium per TiO_2_. These results encourage further study on developing an ideal nanostructure of this new promising anode material for NIBs and exploring their Na-ion storage mechanism.

Herein, we describe a general and facile synthesis route of iso-oriented TiO_2_ mesocages with tunable microstructures and nanoporous nature and their application as high-performance anode in rechargeable Na-ion batteries. It’s notable that the obtained TiO_2_ mesocages are built by very tiny nanocrystals with a mutual orientation, holding a fine nanoporous structure and large surface area. The obtained iso-oriented TiO_2_ mesocages exhibited large capacity and good cycling stability for NIBs. The sodium ions storage is mainly controlled by the pseudocapacitive process, which facilitates the high-rate capability.

## Results

**Figure 1:**
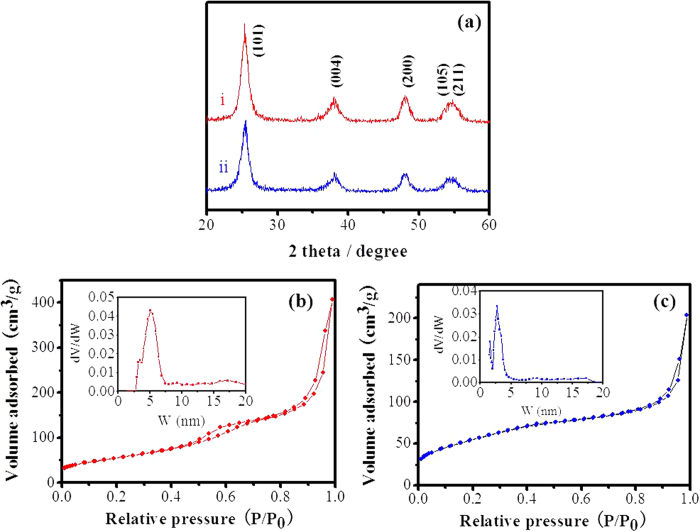
(**a**) XRD patterns of TiO_2_-MN (curve i) and TiO_2_-MC (curve ii), N_2_ adsorption-desorption isotherms of (**b**) TiO_2_-MN and TiO_2_-MC. The insets in (**b**) and (**c**) are the corresponding NLDFT pore size distribution.

The iso-oriented TiO_2_ mesocages were synthesized by a simple and low-temperature route. The samples obtained from HNO_3_ and HCl solution were defined as TiO_2_-MN and TiO_2_-MC, respectivley. Figure 1a shows the related X-ray diffraction (XRD) patterns of as-prepared samples. All the diffraction peaks in Fig. 1 could be exclusively ascribed to tetragonal anatase TiO_2_ (JCPDS 73-1764). The broadened diffraction peaks suggest a small crystallite size of the anatase samples. The average crystallite size of TiO_2_-MN was calculated to be approximately 11 nm (also 11 nm for TiO_2_ -MC), using the Scherer equation, based on the (101) diffraction peak. N_2_ adsorption–desorption isotherms measurements were adopted to reveal the Brunauer–Emmett–Teller (BET) surface area and pore size distribution, as shown in Fig. 1b,c. The BET surface area and the pore volume of TiO_2_-MN were determined to be 204 m^2^ g^−1^ and 0.62 cm^3^ g^−1^, respectively. Morover, TiO_2_-MN exhibits rather uniform nanopores with an average diameter of 5.0 nm in Fig. 1b (inset). Compared to TiO_2_-MC (Fig. 1c), its BET surface area and pore volume were about 199 m^2^ g^−1^ and 0.31 cm^3^ g^−1^, suggesting a lower pore volume and a smaller pore size mostly around 2.7 nm.

**Figure 2:**
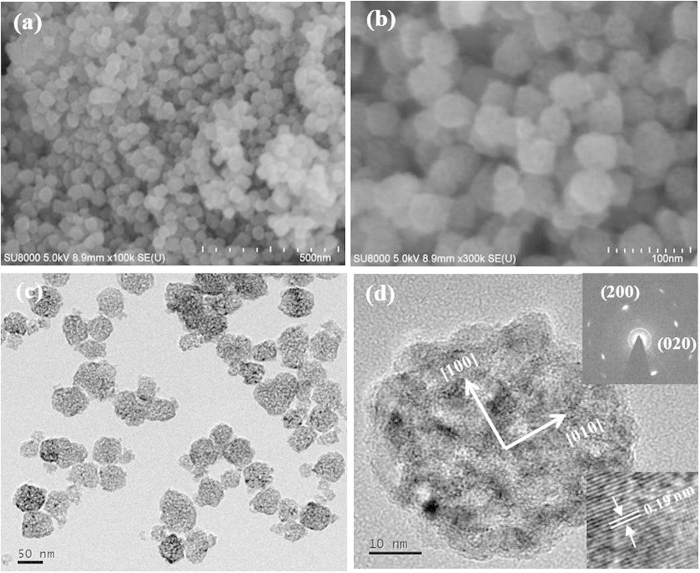
SEM (**a**, **b**) and TEM (**c**, **d**) images of TiO_2_-MN. The insets in (**d**) are the related SAED pattern and HRTEM image, respectively.

Figure 2a,b show the low-magnification and high-magnification SEM image of the sample obtained from 2 M HNO_3_ aqueous solution (TiO_2_-MN), indicating the large-scale formation of nanoparticles with a size of about 30–50 nm. The rough surface and porous nature of the nanoparticles could also be clearly observed (Fig. 2b). The TEM image shown in Fig. 2c further reveals the porous structure of TiO_2_-MN. Figure 2d presents a typical TEM image of a single nanoparticle, which confirms that the anatase TiO_2_ nanoparticle was constructed from tiny nanoparticle subunits with diameter about 5 nm and has a cage-like morphology with nanoporous structure. As shown in the upper inset of Fig. 2d, the SAED pattern taken from the whole nanoparticle exhibited single-crystal-like diffraction, suggesting that the tiny nanoparticle subunits were highly ordered and oriented along the [100] direction. The HRTEM image (inset of Fig. 2d) further confirms that the primary nanocrystals were highly crystalline, the clear lattice fringe of 0.19 nm was assigned to the (200) spacing of anatase structure. It should be pointed that similar single-crystal-like TiO_2_ mesocages were synthesized by Lu *et al.*^27^ through a hard-template (SBA-15) method and demonstrated excellent photocatalytic activity. Herein, the iso-oriented TiO_2_ mesocages with high surface area and uniform mesopores, constructed by highly oriented tiny nanocrystals, were successfully prepared by a facile and low-temperature route.

**Figure 3:**
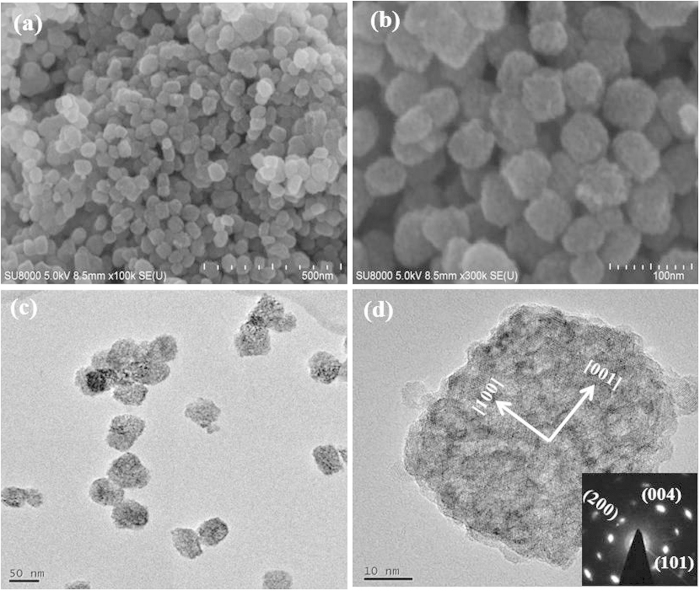
SEM (**a**, **b**) and TEM (**c**, **d**) images of TiO_2_-MC. The inset in (**d**) is the related SAED pattern from the whole nanoparticle.

It’s notable that the iso-oriented TiO_2_ mesocages with tunable microstructures could be prepared by using different anion in this synthesis method. SEM and TEM images of TiO_2_-MC obtained from 2 M HCl aqueous solution are shown in Fig. 3. It is clearly shown that numerous nanoparticles with uniform size (40–60 nm) were formed. TiO_2_-MN possessed of a rough surface, actually, it was constructed by tiny nanoparticle subunits (about 3–5 nm). The corresponding SAED pattern in the inset of Fig. 3d for the whole nanoparticle with single-crystal-like diffractions indicates that the building of nanoparticle subunits were highly ordered, leading to the formation of a crystallographically oriented architecture along [001] direction. Such architecture was named mesocrystal by Cölfen *et al.* which is porous quasi-single crystal consisting of ordered assemblies of aligned nanoparticles ^28^. Furthermore, the diffraction spots were slightly elongated (similar results were observed for TiO_2_-MN), indicating that there was a small mismatch between the boundaries of the nanoparticle subunits; this is usually found for the growth of many ordered nanoparticle assembles (mesocrystals) when they are assembled in the same orientation^28^,^29^.

To shed light on the formation mechanism of the TiO_2_ mesocages, a series of samples were synthesized for different periods of time and the results are shown in Figure S1. It could be observed that some irregular particles were constructed by the tiny anatase TiO_2_ nanocrystals, the boundaries among the nanocrystals were clear and they aggregated together by perfect or imperfect attachments. It’s demonstrated that aggregation of nanocrystals with the same crystallographic orientations, as well as the presence of defects, are strong evidence of oriented attachment (OA)^30^,^31^. This growth mechanism is also a typical formation process for the ordered nanoparticle superstructures (mesocrystals)^28^. Besides the anion (NO_3_^−^ and Cl^−^), the amount of the SDS significantly affect the morphology of the final samples, more SEM images were shown in Figure S2. Therefore, the microstructures of the obtained TiO_2_ mesocages depend on the cooperative effect of the surfactant and anion. It was pointed out that the organic additive could be in favor of the temporary stabilization of the primary nanocrystals, allowing their attachment and assembly into ordered aggregates^28^,^29^. The driving force for the oriented self-assembly could be assigned to the minimization of the total surface energy of disordered primary nanocrystals^28^.

**Figure 4:**
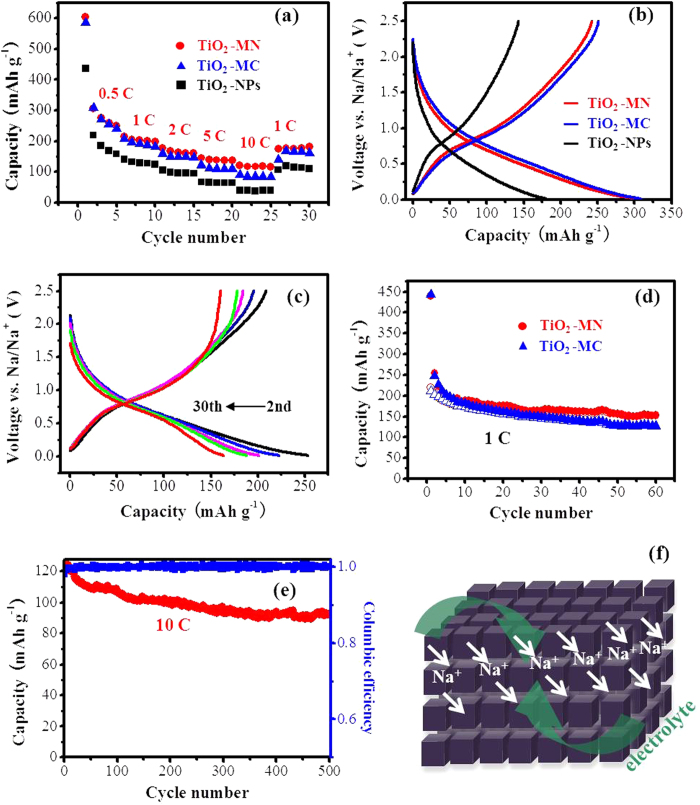
(**a**) Rate capability of TiO_2_-MN, TiO_2_-MC and TiO_2_ nanoparticles from 0.2 to 5 C, (**b**) charge-discharge profiles of the three samples at a current rate of 0.5 C, (**c**) charge-discharge profiles of TiO_2_-MN, (**d**) cycling performance of TiO_2_-MN and TiO_2_-MC (filled symbols: discharge capacity and open symbols: charge capacity), (**e**) long cycling performance of TiO_2_-MN, (**f**) schemes of the electrochemical reaction process of TiO_2_ mesocages.

Finally, the sodium-ion storage properties of the iso-oriented TiO_2_ mesocages with mesoporous structure were investigated. Cells made from commercial anatase TiO_2_ nanoparticles (TiO_2_-NPs) were also fabricated as a reference sample. The XRD, SEM image and N_2_ adsorption-desorption isotherms of TiO_2_-NPs were shown in Figure S3. The BET surface area and the pore volume of TiO_2_-NPs were determined to be 118 m^2^ g^−1^ and 0.32 cm^3^ g^−1^, respectively. Figure 4a shows the rate capability of TiO_2_-MN, TiO_2_-MC and TiO_2_-NPs from 0.2 to 5 C (1C = 170 mAg^−1^). TiO_2_-MN exhibited good rate performance, with a stable capacity of 240 mAhg^−1^ at 0.5 C, 200 mAhg^−1^ at 1 C, 165 mAhg^−1^ at 2 C, and 137 mAhg^−1^ at 5 C, 120 mAhg^−1^ at 10 C. Furthermore, a reversible capacity of 180 mAhg^−1^ could be remained when the current rate returned to 1 C. TiO_2_-MC showed comparable performance at low current rates, but was not so good as TiO_2_-MN at high current rates which may be due to the lower pore volume and smaller pore size. However, both TiO_2_-MN and TiO_2_-MC exhibited superior rate performance compared to TiO_2_ nanoparticles. It displays a stable discharge capacity of 160 mAhg^−1^ at 0.5 C, 125 mAhg^−1^ at 1 C, 97 mAhg^−1^ at 2 C, and 64 mAhg^−1^ at 5 C, 38 mAhg^−1^ at 10 C. Figure 4b shows typical charge-discharge profiles of the three samples at the second cycle, the slope profiles are similar to the previous reports of anatase TiO_2_ as anode material for Na-ion batteries^22^,^23^,^24^,^25^. The character of the charge-discharge profiles was not apparently changed during cycling (Fig. 4c). Figure 4d presents the cycling performance of TiO_2_-MN and TiO_2_-MC, the capacity of 152 mAhg^−1^ and 128 mAhg^−1^ could be remained, respectively, after 60 cycles at 1 C. In order to test the long cyclic stability at high rates, a sodium-ion cell made from TiO_2_-MN were run at 10 C for 500 cycles, after aging at 0.5 C for 5 cycles. Figure 4e shows the capacity starts at 123 mAhg^−1^ and still maintains at 93 mAhg^−1^ after 500 cycles as well as high columbic efficiency, indicating a good cycling ability. Thus, anatase TiO_2_ mesocages exhibit a larger capacity and better rate performance than that of layerd titanante nanotubes and nanorods^32^,^33^. It is suggested that (as the scheme in Fig. 4f) the crystallographically oriented anatase mesocages (composed of tiny nanocrystal subunits) were well connected with few grain boundaries compared with the irregularly oriented nanoparticles, which facilitated the fast Na^+^ ion and electron transport. Moreover, the very large surface area and nanoporous nature in the mesocages could provide a high level of accessibility for the electrolyte and more active sites, and hence allow the efficient ion transport as well as the freedom for volume change.

**Figure 5:**
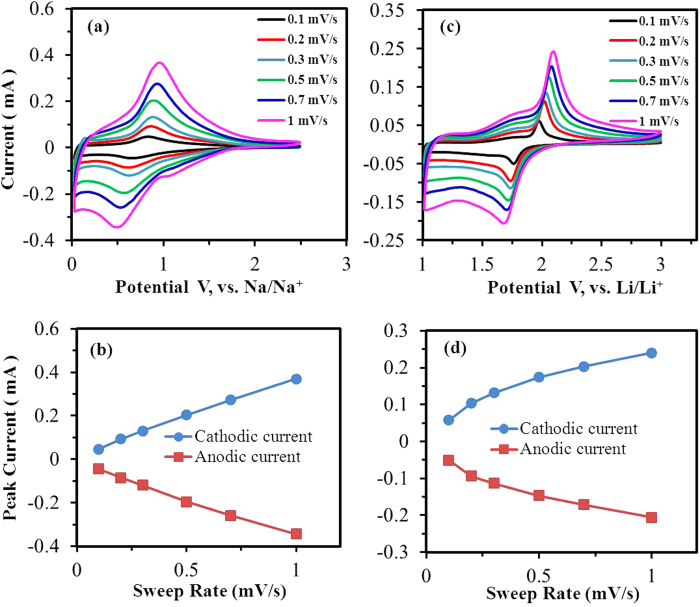
Cyclic voltammetry curves at various sweep rates (**a**, **c**) and the relationship between peak current and sweep rate of TiO_2_-MC (**b**, **d**): (**a**, **b**) sodium-ion battery, (**c**, **d**) lithium-ion battery.

Cyclic voltammetry (CV) experiments performed at various sweep rates (0.1–1 mV/s) were used to examine the redox processes occurring in the as-prepared iso-oriented TiO_2_ mesocages. As shown in Fig. 5a, a couple of redox peaks are observed between 0.4 V and 0.9 V vs. Na/Na^+^, which could correspond to the insertion/extraction of Na-ion. The voltammetric response of electrode active material with respect to sweep rate can be calculated according to^34^:


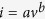


where *i* is the current density, *v* is the scan rate, and a and b are adjustable parameters. When the b-value approaches 1, the electrochemical process is mainly controlled by capacitance, and when the b value is close to 0.5, the insertion behavior (battery behavior) dominates. The pseudocapacitive behavior of electrode material arises from the surface faradic redox reactions, and the insertion behavior is controlled by bulk diffusion process. However, both of them displays obvious redox peaks on CV measurements in a specific voltage region. Figure 5b shows the relationship between peak current and sweep rate on NIBs. As the b-value approaches 1 (0.89 calculated from Fig. 5b), the sodium-ion storage of TiO_2_ mesocages is mainly controlled by the pseudocapacitive process, leading to a fast Na^+^ insertion/extraction and extended cycling life. Rajh *et al.*^21^ also found the pseudocapacitive characteristic of the amorphous TiO_2_ nanotube anode for rechargeable NIBs. However, the lithium-ion storage of the amorphous TiO_2_ nanotube anode is controlled by capacitive process as the discharge voltage was below 1.8 V. In this study, a couple of typical redox peaks are observed from Fig. 5c, corresponding to the insertion/extraction of Li-ion. The b-value approaches 0.5 (0.58 calculated from Fig. 5d), demonstrating the typical insertion behavior of lithium storage in TiO_2_ mesocages. Thus, the Na^+^ and Li^+^ ion storage performance of TiO_2_ mesocages is mainly controlled by pseudocapacitive behavior and insertion behavior, respectively.

**Figure 6:**
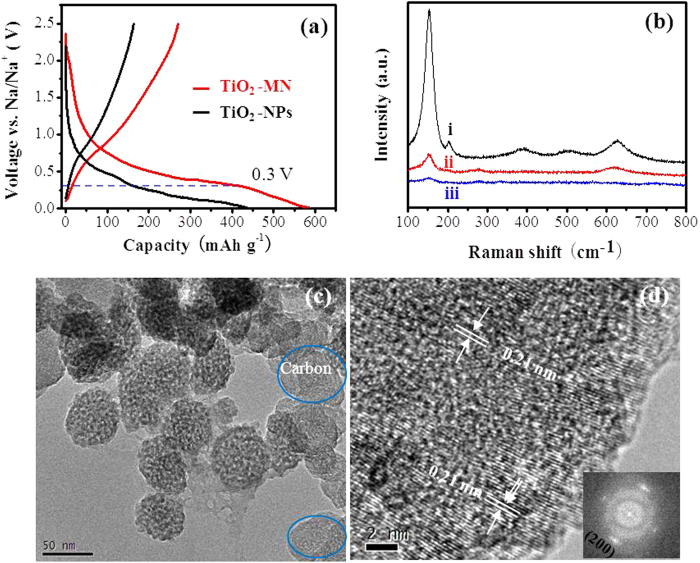
(**a**) The first charge-discharge profiles of TiO_2_-MN and TiO_2_-NPs, (**b**) Raman spectra obtained from TiO_2_-MN electrode at various states: i) fresh, ii) first discharge and iii) first charge, (**c**, **d**) TEM and HRTEM images of TiO_2_ mesocages electrode after rate performance. The inset in (**d**) is the related FFT pattern.

Recently, it’s reported that anatase TiO_2_ nanocrystals displayed a large irreversible capacity at the first cycle especially when the discharge voltage was below 0.3 V^35^,^26^. This large irreversible capacity could be due to occurrence of side reactions with electrolyte, a solid−electrolyte interface (SEI) formation and some irreversible electrochemical reaction of the active materials^25^,^26^,^35^. This phenomenon was also found in the Na_2_Ti_3_O_7_ anode material for sodium-ion batteries^15^,^16^. Similar results were also found in this study, as shown in Fig. 6a. The TiO_2_ nanoparticles (TiO_2_-NPs) exhibited a discharge capacity of 436.3 mAhg^−1^ and charge capacity of 163.5 mAhg^−1^ at 0.5 C at the first cycle, indicating a low columbic efficiency of 37.4%. Besides, a large irreversible capacity of about 284 mAhg^−1^ was observed under 0.3 V. However, TiO_2_ mesocages displayed a large discharge and charge capacity of 586 mAhg^−1^ and 270 mAhg^−1^, and thus higher columbic efficiency of 46.1%. Moreover, the irreversible capacity (180 mAhg^−1^) under 0.3 V is also smaller than that of TiO_2_-NPs. This could be due to the crystallographically oriented mesocages with few grain boundaries compared with the irregularly oriented nanoparticles, leading to less irreversible reaction and fast electron transport.

It’s well known that Raman spectroscopy is an effective measurement to investigate the structure of titanium dioxide, and the results are shown in Fig. 6b. The curve of fresh electrode exhibits the typical Raman peaks of anatase TiO_2_^36^. During the discharge and charge process, the Raman spectra of the electrode suggest that the crystallinity of the anatase TiO_2_ continuously weakens, which is in agreement with the results reported by Wu *et al.*^26^ However, the morphology of the TiO_2_ mesocages remarkably remained unchanged even after different rate cycling, as displayed in Fig. 6c,d shows the HRTEM image and the related FFT pattern of TiO_2_ mesocages after rate performance at full charge state, the whole particle was not well crystalline as before, which is in agreement with the Raman results. In spite of a relatively low crystallinity of the anatase TiO_2_ mesocages, the lattice fringe of 0.21 nm could be clearly observed from the crystalline area, indicating a slight increase in the d-spacing distance during the repetition of Na^+^ insertion and extraction. It’s worth mentioning that the tiny nanoparticle subunits in TiO_2_ mesocages still keep oriented along the [100] direction after Na^+^ insertion and extraction. This result is different from the reports of Kim *et al.*, which demonstrated reversible variation of the lattice of anatase TiO_2_ nanorods even after 100 cycles^25^. It’s suggested that Na^+^ insertion into anatase single crystals is not easy, and a low crystallinity as well as an increase in lattice may provide a lower energy barrier for Na^+^ insertion. Herein, the anatase mesocages composed of highly oriented tiny nanocrystal subunits with large surface area and porous nature of mesocages could provide more active reaction sites for Na^+^ storage and a good freedom for volume change during Na^+^ insertion.

## Discussion

Iso-oriented TiO_2_ mesocages with mesoporous nature and tunable microstructures were successfully fabricated via a facile synthesis route. The TiO_2_ mesocages were used for the first time as anode materials in rechargeable Na-ion batteries, demonstrating a large reversible charge-discharge capacity, good rate capability and cycling performance. This could be attributed to the intrinsic characteristics of the mesocages constructed by crystallographically oriented nanoparticle subunits with few grain boundaries compared with the irregularly oriented nanoparticles, accompanied by a large surface area and uniform nanoporous nature. Such architecture facilitated fast electron and ion transport, and gave more active sites and freedom for volume change for Na-ion insertion. Moreover, the sodium-ion storage process of anatase mesocages was mainly controlled by pseudocapacitive behavior, leading to high rate performance and making them promising for applications in rechargeable Na-ion batteries.

## Methods

### Materials Synthesis

All chemicals were purchased from Aladdin without further purification. In a typical synthesis, 1.5 g sodium dodecyl sulfate (SDS) was first dissolved in 50 mL 2 M HNO_3_ or HCl solution. After the solution was stirred for a few minutes, 1 mL (1.5 mL for HCl solution) of titanium (IV) isopropoxide (TIP) was added and kept at 70 °C (80 °C for HCl solution) for 48 h under stirring. The final products were obtained by centrifugation, washed thoroughly with distilled water and dried at 60 °C overnight, and then calcined at 400 °C for 30 min in air to remove the residual organics. The samples obtained from HNO_3_ and HCl solution were denoted as TiO_2_-MN- and TiO_2_-MC, respectively.

### Characterizations of the samples

Scanning electron microscopy (SEM, S8010 instrument) and Transmission electron microscopy (TEM, FEI F20 S-TWIN instrument) were applied for the structural characterization of the resulting nanowires and mesocrystals. X-ray diffraction (XRD) patterns were recorded on a Rigaku X-ray diffractometer using Cu Kα (λ = 1.5418 Å) radiation. N_2_ adsorption-desorption analysis was measured on a Micro-meritics TriStar II 3020 instrument (USA). The pore size distributions of the as-prepared samples were analyzed using the Non-Local-Density Functional Theory (NLDFT) methods. The Raman spectra were recorded on a LabRAM HR Evolution (HORIBA Jobin Yvon) with a 532 nm laser.

### Electrochemical Measurements

For the electrochemical measurement of Na-ion intercalation, TiO_2_ mesocages were admixed with polyvinylidene fluoride (PVDF) binder and acetylene black carbon additive in a weight ratio of 70:20:10. The mixture was spread and pressed on copper foil circular flakes as working electrodes (WE), and dried at 120 °C in vacuum for 12 h. Na-ion cells were assembled in coin-type cells (CR 2025) with a Na metal foil as the negative electrode, glass fiber separator (Whatman GF/F), and 1 M NaClO_4_ in ethylene carbonate (EC) and diethyl carbonate (DEC) (1/1 in volume) as the electrolyte. The cells were assembled in a glove box filled with highly pure argon gas (O_2_ and H_2_O levels < 1 ppm), and charge/discharge tests were performed in the voltage range of 0.01 to 2.5 V (Na^+^/Na) on a Land automatic batteries tester (Land CT 2001A, Wuhan, China). The active material content in the electrode was around 1.2 mg cm^−2^, and the amount of electrolyte was 170–200 μL. The cells were tested under a constant temperature (25 °C). Cyclic voltammetry (CV) measurements were performed on Zennium (Zahner).

## Additional Information

**How to cite this article**: Hong, Z. *et al.* Iso-Oriented Anatase TiO_2_ Mesocages as a High Performance Anode Material for Sodium-Ion Storage. *Sci. Rep.* **5**, 11960; doi: 10.1038/srep11960 (2015).

## Supplementary Material

Supplementary Information
