# Supplementary material for: Iso-Oriented Anatase TiO2 Mesocages as a High Performance Anode Material for Sodium-Ion Storage
Source: Sci Rep. 2015 Jul 6;5:11960. doi: 10.1038/srep11960 (PMC4491717; doi:10.1038/srep11960)
Supplement: Supplementary Information [file srep11960-s1.pdf]

Supplementary Information

Iso-Oriented Anatase TiO_2 Mesocages as a High Performance Anode Material for Sodium-Ion Storage

Zhensheng Hong,[a] Kaiqiang Zhou, [a] Zhigao Huang, [a] Mingdeng Wei [b,c]

[a] Fujian Provincial Key Laboratory of Quantum Manipulation and New Energy Materials, College of Physics and Energy, Fujian Normal University, Fuzhou, Fujian 350108, China. [b] State Key Laboratory of Photocatalysis on Energy and Environment, Fuzhou University, Fuzhou, Fujian 350002, China. [c] Institute of Advanced Energy Materials, Fuzhou University, Fuzhou, Fujian 350002, China; Correspondence and requests for materials should be addressed to Z.S.H.(winter0514@163.com) or M.D.W.(wei-mingdeng@fzu.edu.cn).

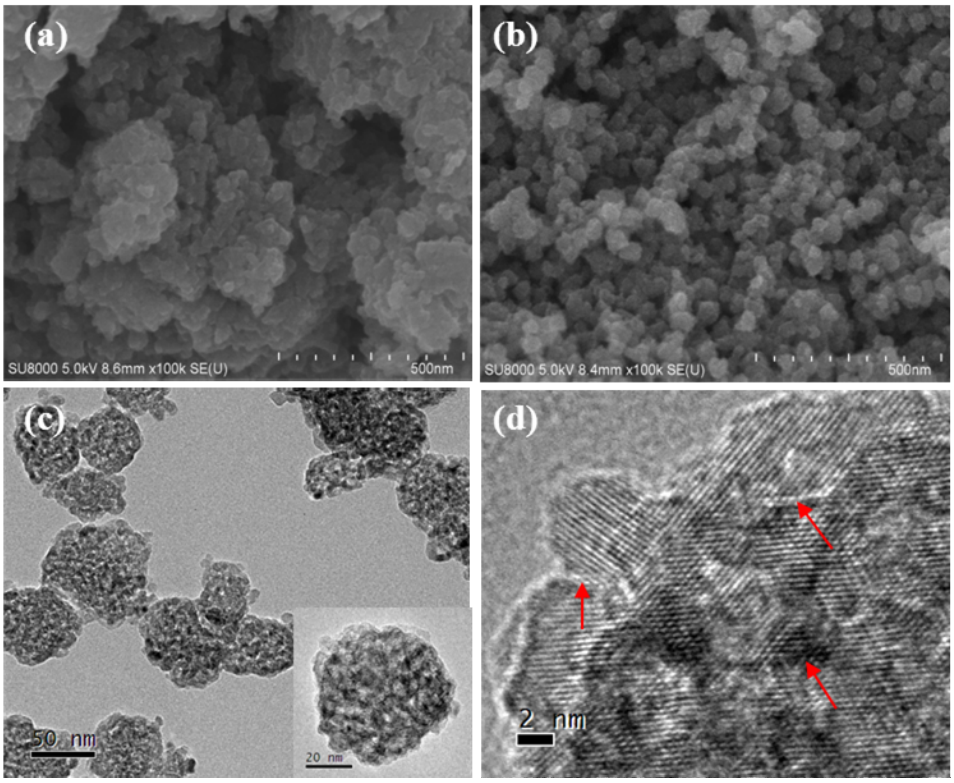

Supplementary Figure S1 | SEM (a, b), TEM (c) and HRTEM (d) images of samples obtained under different reaction times: (a) 12 h and (b-d) 24 h. The inset in (c) is a single irregular particle.

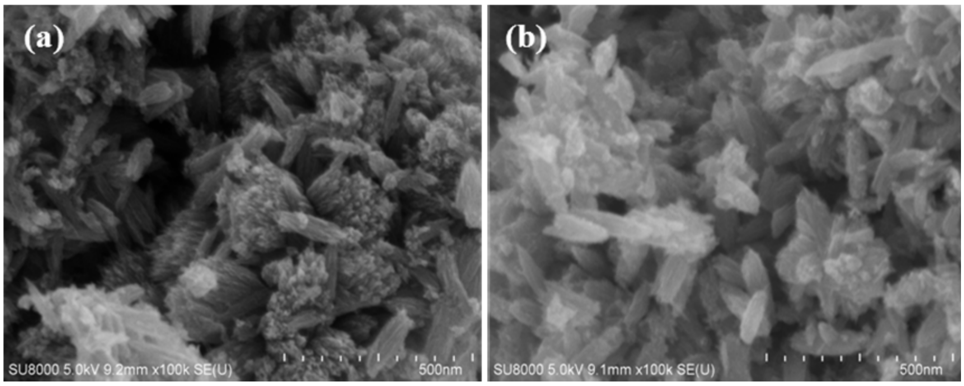

Supplementary Figure S2 | SEM images of samples obtained under different amount of SDS: (a) 0.5 g and (b) 1 g.

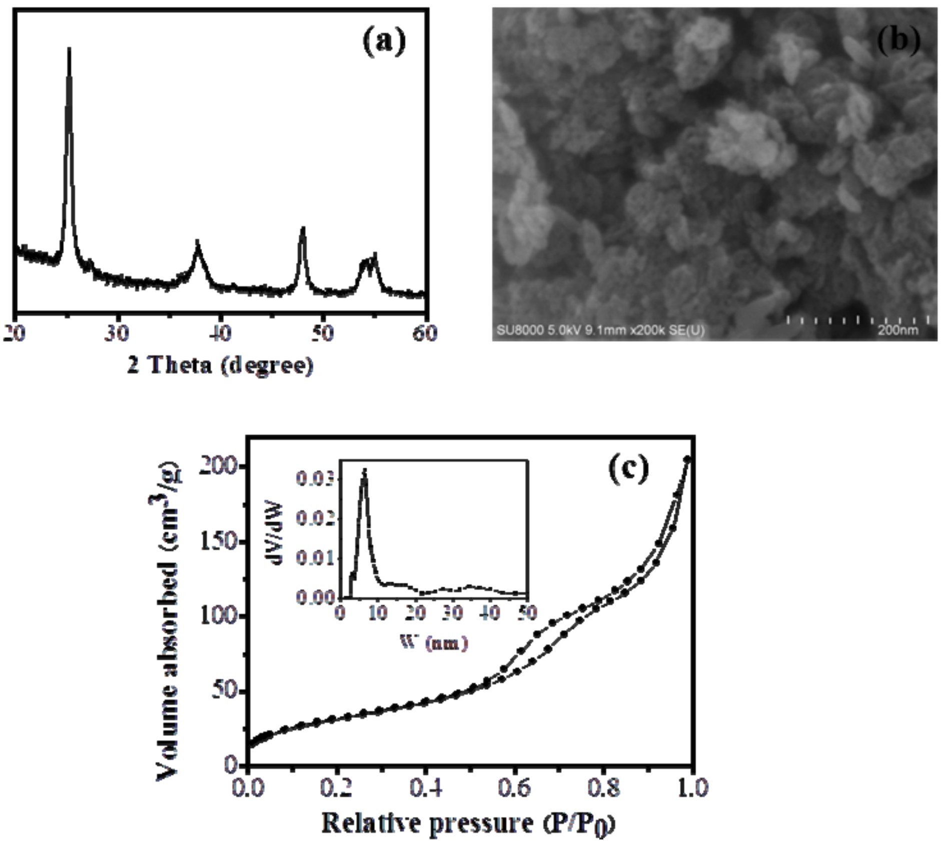

Supplementary Figure S3 | (a) XRD patterns, (b) SEM image, (c) N_2 adsorption-desorption isotherms of TiO_2 commercial nanoparticles. The inset in (c) is the corresponding NLDFT pore size distribution.

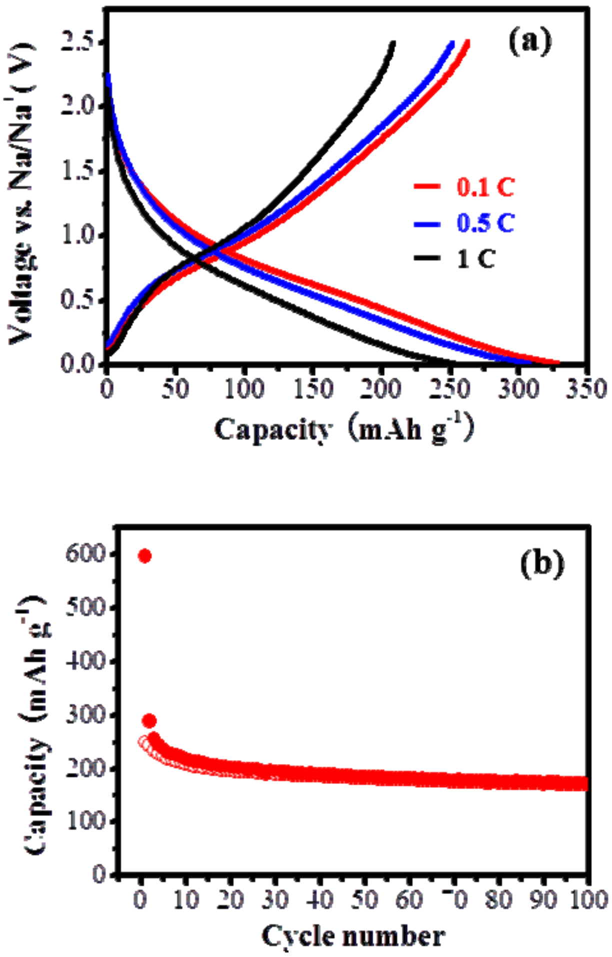

Supplementary Figure S4 | (a) Charge-discharge profiles of TiO_2-MN at different current rate and (b) cycling performance of TiO_2-MN at 0.5 C (filled symbols: discharge capacity and open symbols: charge capacity).

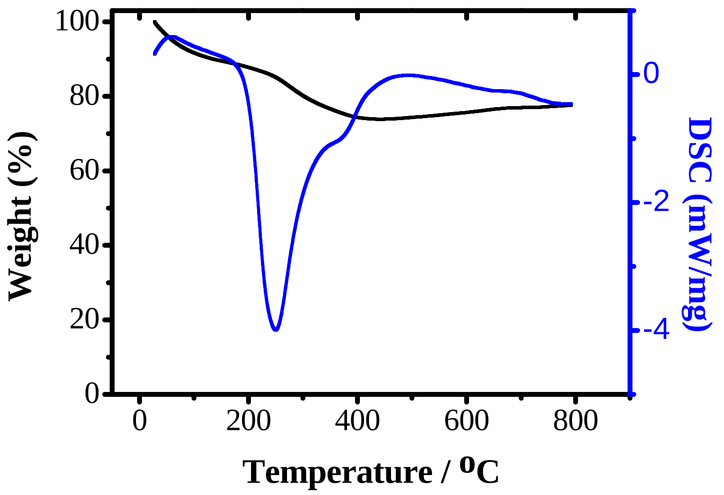

Supplementary Figure S5 | TG and DTA curves of the fresh TiO_2-MN before calcination.
